# Self-Reported Body Awareness: Validation of the Postural Awareness Scale and the Multidimensional Assessment of Interoceptive Awareness (Version 2) in a Non-clinical Adult French-Speaking Sample

**DOI:** 10.3389/fpsyg.2022.946271

**Published:** 2022-07-26

**Authors:** Lucie Da Costa Silva, Célia Belrose, Marion Trousselard, Blake Rea, Elaine Seery, Constance Verdonk, Anaïs M. Duffaud, Charles Verdonk

**Affiliations:** ^1^Department of Neurosciences and Cognitive Sciences, Unit of Neurophysiology of Stress, French Armed Forces Biomedical Research Institute, Brétigny-sur-Orge, France; ^2^Traduction Édition Scientifique, Toulouse, France; ^3^Department of Cardiac Surgery and Cardiology, Bichat-Hospital, Paris, France

**Keywords:** body awareness, proprioception, interoception, PAS, MAIA-2

## Abstract

Body awareness refers to the individual ability to process signals originating from within the body, which provide a mapping of the body’s internal landscape (interoception) and its relation with space and movement (proprioception). The present study aims to evaluate psychometric properties and validate in French two self-report measures of body awareness: the Postural Awareness Scale (PAS), and the last version of the Multidimensional Assessment of Interoceptive Awareness questionnaire (version 2, MAIA-2). We collected data in a non-clinical, adult sample (*N* = 308; 61% women, mean age 35 ± 12 years) using online survey, and a subset of the original sample (*n* = 122; 62% women, mean age 44 ± 11 years) also completed the retest control. Factor analyses and reliability analyses were conducted. Construct validity of the PAS and the MAIA-2 were examined by testing their association with each other, and with self-report measures of personality (Big Five Inventory), alexithymia (Toronto Alexithymia Scale) and dispositional trait mindfulness (Freiburg Mindfulness Inventory). Factor analyses of the PAS supported the same two-factor structure as previously published versions (in other languages). For the MAIA-2, factor analyses suggested that a six-factor structure, excluding Not-Worrying and Not-Distracting factors, could successfully account for a common general factor of self-reported interoception. We found satisfactory internal consistency, construct validity, and reliability over time for both the PAS and the MAIA-2. Altogether, our findings suggest that the French version of the PAS and the MAIA-2 are reliable self-report tools to assess both components of body awareness (proprioception and interoception dimension, respectively).

## Introduction

The investigation of how the brain perceives the body has increased considerably in the past decade, particularly in clinical neuroscience. Indeed, disrupted body awareness is prominently featured in the diagnosis of a wide range of diseases encompassing physical (e.g., chronic pain; Van Der Maas et al., 2016) and mental disorders (e.g., anxiety, eating disorders, etc.; Khalsa et al., 2018). In parallel, body-centered practices (e.g., mindfulness-based programs, meditation, etc.) are increasingly investigated with a mechanistic focus on how they might improve mental health and well-being, in particular through enhanced awareness of bodily signals (Farb et al., 2015; Treves et al., 2019).

Body awareness has been defined (and operationalized) as a psycho-cognitive construct that refers to the individual ability to feel engaged by information coming from the body and noticing subtle changes (Mehling et al., 2009). From a neural perspective, bodily signals continuously provide the brain with a mapping of the body’s internal physiological state (interoception), and with information about the relation the body has with space and movement (proprioception). Interoception entails the integrative interpretation of a variety of stimuli (e.g., signals from the heart, humoral receptors, and free nerve endings)—in a cognitive/emotional context—to derive an overall physiological representation of the state of the body, including conscious and nonconscious aspects (Craig, 2002; Berntson and Khalsa, 2021). On the other hand, proprioception is made up of signals from various peripheral receptors (e.g., somatosensory and vestibular receptors) that are integrated at the central level to provide representation of the body’s orientation relative to gravity (Tuthill and Azim, 2018), which in turn contributes to postural control (Forbes et al., 2018). Of note, postural control relies on cerebral processes that mostly operate unconsciously, but individuals may be partially aware of action of postural balance and can volitionally control it when desired (Amboni et al., 2013; Forbes et al., 2018).

For some authors, the construct of body awareness may be considered as a trait-like characteristic since “the view one has regarding one’s body and bodily processes are likely to influence the way persons experience themselves” (Fisher and Cleveland, 1958; Rani and Rao, 1994; Ferentzi et al., 2020, p. 2). This consideration is strengthened by the idea that an innate and primitive form of body awareness could exist at birth and allow the newborns to integrate primitive sensations such as interoceptive, proprioceptive and vestibular feelings (pleasure/pain and relaxation/tension). According to Riva (2018), bodily experiences characterize the childhood development of the self, through six forms of representations: the minimal selfhood (feel the body like a separate structure from the outside world), the self-location (bodily representation in space), the active body (recognize and actuate bodily actions), the personal body (the first-person experience), the objectified body (the knowledge of being exposed and visible to others), and the social body (the body perception generated by body-related narratives and directed by social norms such as the ideal body). This link between this inborn body awareness and the construction of the self-perception, suggests the existence of a personal body awareness associated with a unique way of perceiving the body, the space around it and the way we react to it through our motor and bodily reactions which are an important part of our personality (Riva, 2018). In that respect, it has been suggested that body awareness could be associated with major dimensions of personality, as measured with the Big Five Inventory (John et al., 1991; Goldberg, 1993). In line with this theoretical suggestion, some studies reported significant association between interoception and personality dimensions of Openness and Conscientiousness (Trapnell and Campbell, 1999; Ferentzi et al., 2017, 2020). The Openness dimension is used to characterize original, imaginative and curious people (Costa and McCrae, 1992) who feel inspired, interested and determined (Letzring and Adamcik, 2015). Because of their interest in new experiences and sensations, a variety of sensory experiences can be attractive for people with a high level of Openness who are more likely to engage in body-related activities such as physical activity (Wilson and Dishman, 2015; Sutin et al., 2016). Relationships between openness and mindfulness practice, which requires attention to the body, have also been found (van den Hurk et al., 2011) but sometimes only for participants who have already practiced mindfulness (Thompson and Waltz, 2007), suggesting that it is the engagement of these individuals in this type of practice that leads to a greater level of body awareness. With regard to the Conscientiousness dimension, the main characteristic of this personality type is self-discipline (Costa and McCrae, 1992). Indeed, it appears that people with a high level of Conscientiousness have the ability to control themselves and, more specifically, their emotions and behaviors through the use of functional regulation strategies (Jensen-Campbell et al., 2007) which requires the ability to direct attention to the bodily sensations caused by unpleasant emotions in order to manage and overcome them. In this view, we can suppose that these two dimensions of the personality are associated with a better body awareness. Yet, it should be noted that relationship between interoception and personality has not been reported systematically (Sze et al., 2010), and did not encompass all personality dimensions, e.g., body awareness was reported to be independent of the dimension of Neuroticism (Shields et al., 1989; Ferentzi et al., 2017, 2020). In contrast to Conscientiousness, neuroticism is characterized by poor emotional regulation strategies, which may explain that people with high levels of neuroticism are more sensitive to stress than others (Costa and McCrae, 1992) and experience more negative affect (Gross et al., 1998). This difficulty in regulating emotions has also been found in people with alexithymia (Swart et al., 2009), which is a disorder leading to difficulties in describing feelings and distinguishing emotions from bodily sensations (Taylor, 1984; Sifneos, 1991). In the case of a personality with a high level of neuroticism, as in the case of an alexithymia disorder, that implies poor interoception skills. Indeed, emotional feeling states arise from physiological changes that occur within internal organs, and emotions themselves track and steer the redirection of physiological resources to adapt behavior (Critchley and Garfinkel, 2017). Moreover, it has been shown that alexithymia is associated with deficit in interoception (as assessed with heartbeat perception tasks into which participants are instructed to report either the number or the timing of their heartbeats; Herbert et al., 2011; Murphy et al., 2018a,b).

Paralleling findings from clinical science, recent contemplative research suggests that body awareness is fundamental for adaptive behavior and is intimately connected to self-regulation and homeostasis (Farb et al., 2015). Contemplative practice, such as mindfulness meditation, relies on training the mind to pay sustained attention to the current body experience, primarily the breath, and deliberately returning attention to it whenever distracted (Lutz et al., 2015). Indeed, it can be argued that the more fully an individual is apprised of what is occurring within one’s body, the more adaptive and value consistent the individual’s behavior is likely to be. Previous studies have shown that trait mindfulness, i.e., individual differences in the ability to be mindful in daily life that are supposed to be relatively stable over time (Brown and Ryan, 2003), is associated with enhanced interoception (Mehling et al., 2012; Hanley et al., 2017; Verdonk et al., 2021) and proprioception (Cramer et al., 2018; Topino et al., 2020). In addition, body-centered interventions (e.g., contemplative training) was reported as increasing self-reported interoception, as well as interoceptive accuracy in heartbeat perception tasks (Bornemann et al., 2015; Bornemann and Singer, 2017).

Signals coming from the inside and the outside of the body (body awareness) are usually measured through a range of different tools: experimental tasks and self-report instruments. Furthermore, in the last decade, neuroimaging studies have implemented those measures together in order to explore the brain areas supporting the integration of information to build up the sense of bodily awareness (Salvato et al., 2020). The rubber hand illusion paradigm, based on visuotactile mismatched information, is the most famous experimental task to measure exteroceptive information. This task relies on a perceptual illusion in which the integration of artificial limbs into the body representation of a person lies on combined visual and tactile stimulation. Inside signals have been investigated through the integration of various body sensation state of the internal body and its visceral organs; In particular, heart beats perception paradigm in which participants have to count the number of times they perceive their heart beating during a period of time (Garfinkel et al., 2015). Secondly, body awareness can also be assessed using subjective measures such as self-report questionnaires. To our knowledge, there is currently no psychometric tool validated in French that enables assessment of the proprioceptive dimension of body awareness. Interestingly, Cramer et al. (2018) have developed the Postural Awareness Scale (PAS; Cramer et al., 2018), which was recently validated in Italian (Topino et al., 2020) and in English (Colgan et al., 2021). Furthermore, regarding the interoceptive dimension of body awareness, only the first version of the Multidimensional Assessment of Interoceptive Awareness questionnaire (MAIA-1; Mehling et al., 2012) has been very recently validated in a French-speaking sample (Willem et al., 2021). The PAS and the MAIA have the theoretical advantage to specifically assess one of the two main dimensions of body awareness, namely either interoception or proprioception, thus probably contributing to make them more robust than previously developed self-report measures that assess body awareness in a more global fashion (Mehling et al., 2009). Although self-report instruments raise some long-standing methodological concerns (social desirability biases, vulnerability to limitations of introspection, etc.; Baumeister et al., 2007), they remain widely used in the field of neuroscience because they are particularly attractive, especially, but not exclusively, for efficient field research.

In addition, the PAS is the only postural questionnaire that has been developed to capture increases in proprioceptive awareness in subjects after the implementation of a mind–body training program (i.e., yoga; Cramer et al., 2018). This ability of the questionnaire to assess the influence of body-related activities and therapies on proprioception makes it a particularly useful tool in the field of clinical neuroscience. Regarding to interoception tools, most existing self-report questionnaires on interoception either focus on emotionally induced bodily sensations (e.g., the Autonomic Perception Questionnaire, Mandler et al., 1958; the Somatic Perception Questionnaire, Stern and Higgins, 1969), bodily cycles and rhythms (Body Awareness Questionnaire, Shields and Simon, 1991), or have been developed to be adapted to populations suffering from psychopathological disorders (e.g., schizophrenia with Body Awareness Scale; Roxendal, 1985). The MAIA developed by Mehling et al. is unique in that it is the most comprehensive measure of interoceptive awareness in healthy individuals (Mehling et al., 2012, 2018). This is why, in the present study, we aimed to validate in French the PAS and the last version of the MAIA (version 2, MAIA-2) in a non-clinical adult sample. Construct validity was assessed with self-reporting measurements of mindfulness with the Freiburg Mindfulness Inventory (FMI; Walach et al., 2006; Trousselard et al., 2010), personality with the Big Five Inventory (BFI; John et al., 1991; Plaisant et al., 2010) and alexithymia with the Toronto Alexithymia Scale (TAS-20; Bagby et al., 1994a; Loas et al., 1995). We hypothesized good psychometric properties for the PAS and the MAIA-2, including good internal consistency and satisfactory reliability over time. We also expected positive intercorrelation between each other, and positive correlation with the FMI. Because of the emotional regulation difficulties of people with high levels of Neuroticism (Gross et al., 1998) and people with alexithymia (Swart et al., 2009), which can be attributed to poor body awareness, we expect negative correlation with the TAS-20 and the dimension Neuroticism of the BFI. On the contrary, due to the body awareness characteristics that constitute the Openness and Conscientiousness dimensions of personality, we expect to see positive correlations with these dimensions, in line with the links that have been previously found between personality and MAIA scores (Trapnell and Campbell, 1999; Ferentzi et al., 2017, 2020). Finally, we assumed to find a significant effect of several non-psychological factors, such as gender, sport activity and body-centered practices on the scores of the PAS and the MAIA-2.

## Materials and Methods

### Translation Procedure

For the first step of the validation, we followed the international guidelines of cross-cultural adaptation of self-administered questionnaires (Beaton et al., 2000). With the agreement of the original authors (Mehling W.E. and Cramer H.), the questionnaires were translated by native French-speakers [one psychologist (CB), one researcher in the field of neuroscience (AD), and one medical doctor (CV)]. Then, a concertation meeting (with the initial translators CB, AD, and CV) and one additional medical doctor (MT) was conducted in order to harmonize the French translations. Subsequently, the translated questionnaires were back translated by three English speakers totally blind to the original version [one American student in neuroscience (BR), one professional translator (ES), and one naive French speaker with fluency in English (CGV)]. A final harmonization meeting involving translators of the two steps procedure (CB, AD, CV, ES, and CGV) as well as a student in clinical psychology (LDCS), was held in order to come to satisfactory formulations and validate the translation process. French versions of the PAS and the MAIA-2 that were validated in the present study are shown in the Supplementary Material. Of note, we completed the translation process of the MAIA-2 a few months ahead of the publication of the French version of the MAIA-1 (Willem et al., 2021). As a consequence, common items between MAIA-1 and MAIA-2 questionnaires may show slightly different formulations in their French version.

A “field test” was performed with a group of 20 participants to determine whether the translated items of the PAS and MAIA-2 retained the same meaning as the original items. In this pilot testing, each participant completed the two self-questionnaires and was interviewed to probe about what he or she thought was meant by each questionnaire item and the chosen response. The French translation of the PAS and the MAIA-2 has been validated when investigators were sure that there was no linguistic confusion. This process revealed a good understanding of the French translation and no revision was needed to the final translated version of the questionnaires.

### Participants and Data Collection

Our study was conducted online following standards for Internet-based experimenting (Reips, 2002). Participants were recruited through announcements that were posted on different websites and social media. To be eligible for inclusion in the study, a subject had to (i) report no history of neuropsychiatric disease and chronic pain, (ii) be over 18 years and under the age of 65, and (iii) be able to read and understand French. At the beginning of the survey the participants were informed of the aim of the study and consented to participate by clicking the “next” button on the online survey. They also received, *via* email, the study information letter. No compensation was offered for the participation in the study. They were guaranteed privacy and anonymity. The data were collected online *via* the LimeSurvey tool (LimeSurvey Project Team/Schmitz, 2012).^1^

### Measures

The socio-demographic data included age, gender, weight, height, educational level, sport practice (frequency, average duration of sport, body-oriented practice), history of injury which changed body perception and history of chronic pain.

### Questionnaires

#### Postural Awareness Scale

The 12-item Postural Awareness Scale measures two facets of postural body awareness: (1) *Ease/familiarity with postural awareness* (PAS-EwPA): effortless awareness of the body posture and (2) *Need for attention regulation with postural awareness* (PAS-NfA): awareness of the posture requires efforts to balance conscious cognitive processes and bodily needs. The two facets can be interpreted as two opposite ends of a continuum effort necessary to becoming aware of one’s posture (Cramer et al., 2018). The questionnaire is scored using a seven-point scale, with responses ranging from 1 (not like me at all) to 7 (completely like me). For each of the two subscales, the score was counted by adding the rating for all items; items related to the subscale *Need for attention regulation with postural awareness* (items 1, 2, 3, 4, 5 and 12) were reversed beforehand.

#### Multidimensional Assessment of Interoceptive Awareness (Version 2)

The 37-item Multidimensional Assessment of Interoceptive Awareness (MAIA-2) questionnaire, developed by Mehling et al. (2018), measures eight facets of interoceptive body awareness: (1) *Noticing* (MAIA-2-N): awareness of uncomfortable, comfortable, and neutral body sensations; (2) *Not-distracting* (MAIA-2-ND): tendency not to be distracted by oneself from sensations of pain or discomfort; (3) *Not-worrying* (MAIA-2-NW): tendency not to worry with sensations of pain or discomfort; (4) *Attention regulation* (MAIA-2-AR): ability to sustain and control attention to body sensation; (5) *Emotional Awareness* (MAIA-2-EA): awareness of the connection between body sensations and emotional states; (6) *Self-regulation* (MAIA-2-SR): ability to regulate psychological distress by attention to body sensations; (7) *Body listening* (MAIA-2-BL): actively listens to the body for insight; and (8) *Trusting* (MAIA-2-T): experiences one own’s body as safe and trustworthy. The questionnaire is scored using a six-point scale, with responses ranging from 0 (never) to 5 (always). For each of the eight subscales, the score was counted by averaging the scores of items belonging to the subscale (items 5, 6, 7, 8, 9, 10, 11, 12, and 15 were reverse scored). Of note, the MAIA-2 includes five additional items with regard to the version 1 of the MAIA (MAIA-1; Mehling et al., 2012; Willem et al., 2021) that have been added to improve internal consistency and reliability of the MAIA (Mehling et al., 2018).

#### Freiburg Mindfulness Inventory

The 14-item Freiburg Mindfulness Inventory (FMI), developed by Walach et al. (2006) measures dispositional trait mindfulness by indexing facets of Presence (i.e., being aware of all experiences in the present moment) and Non-judgmental acceptance (i.e., understanding that things are not necessarily how one wishes them to be). This questionnaire is semantically independent from a meditation context and it is applicable to all population groups, in particular to those with no practice of mindfulness meditation (Walach et al., 2006; Trousselard et al., 2010). The questionnaire is scored using a four-point scale, with responses ranging from 1 (rarely) to 4 (almost always). In the French version, a total mindfulness score was computed by adding the rating for all items, except for the 13th item which was reversely scored (Trousselard et al., 2010).

#### Big Five Inventory

The 44-item Big Five Inventory (BFI-FR) was used to describe the five main personality traits: (1) E: Extraversion, Energy, and Enthusiasm; (2) A: Agreeableness, Altruism, and Affection; (3) C: Conscientiousness, Constraint, and Control of impulse; (4) N: Neuroticism, Negative affectivity, and Nervousness; and (5) O: Openness, Originality, and Open-mindedness. Each item is rated on a 5-point Likert scale from 1 (disagree a lot) to 5 (agree a lot; Plaisant et al., 2005, 2010).

#### Toronto Alexithymia Scale

The 20-item Toronto Alexithymia Scale (TAS-20) assesses the level of alexithymia (Bagby et al., 1994a,b). It is scored on a 1- to 5-point Likert scale. The questionnaire measures three main dimensions of alexithymia: (1) difficulty in identifying feelings and distinguishing between feelings and bodily sensations in emotional activation (DIF), (2) difficulty in the verbal expression of emotions (DVE), and (3) externally oriented thinking (EOT; Loas et al., 1995; Zimmermann et al., 2007).

### Statistical and Data Analysis

Data analyses were performed using R (version 3.5.3; R Core Team, 2013) and JASP (version 0.11.1).^2^

#### Factor Structure

**Figure 1:**
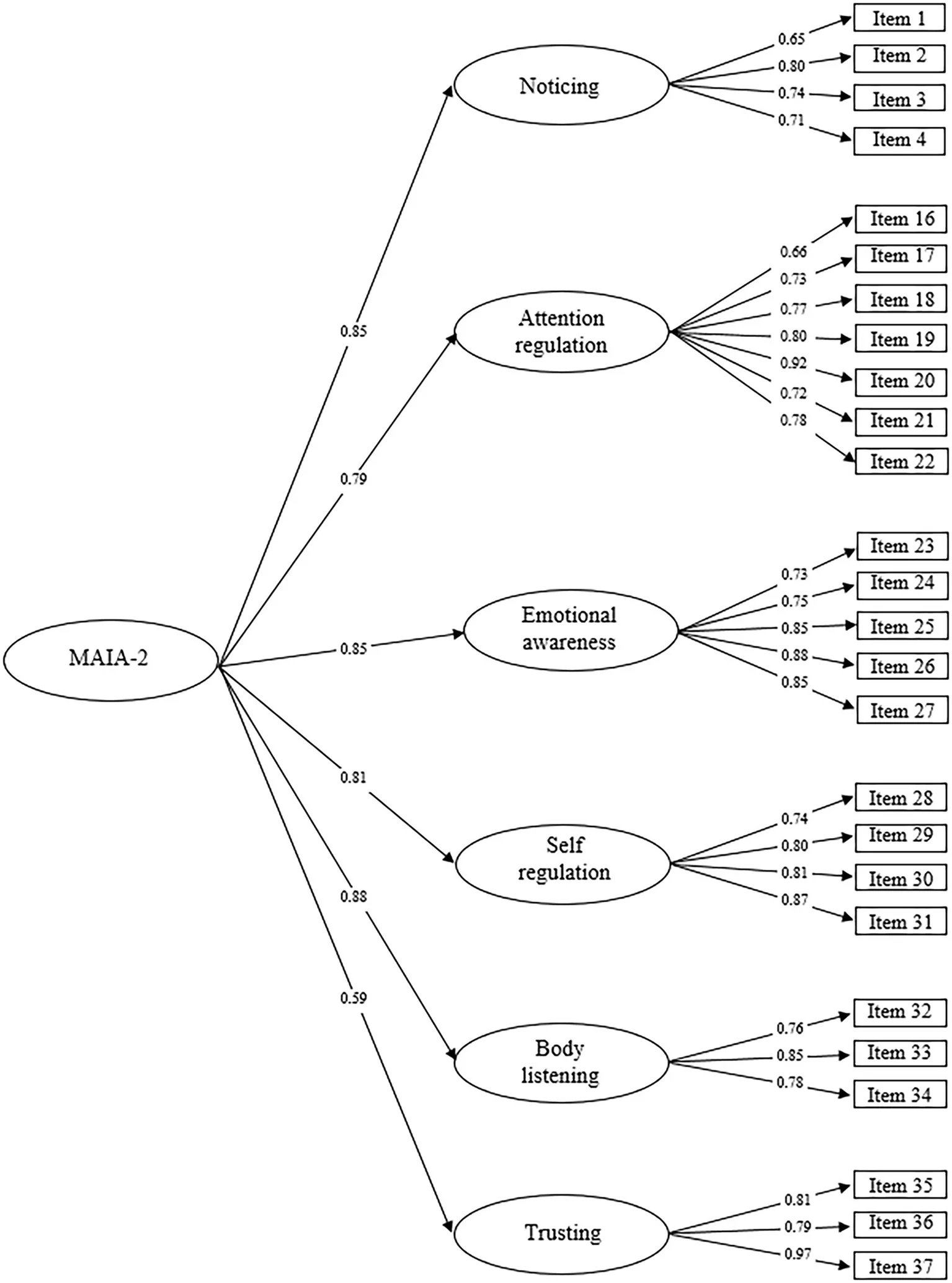
Simplified illustration of the MAIA-2 factor model showing the best model fit based on our data. Specifically, this model excludes the factors Not-distracting and Not-worrying and responses to items related to these two factors have been removed from the dataset. Values presented represent the standardized regression coefficients.

We tested whether the factor structure originally proposed for the PAS (Cramer et al., 2018) and for the MAIA-2 (Mehling et al., 2018) would replicate in the French version. For this purpose, we conducted Exploratory Factor Analysis (EFA) on a subset of the original sample including 50% of the available data (154 subjects). Horn’s parallel analysis (HPA) was performed to determine the optimal number of factors to extract using principal axis factoring and promax rotation (Horn, 1965). Subsequently, Confirmatory Factor Analysis (CFA) were conducted on the remaining 50% of the available data (154 subjects). We tested a higher-order model in which a second-order factor (e.g., the factor *Interoceptive awareness* for the MAIA-2) causes individual differences in several first-order factors (e.g., the subscales *Noticing* and *Trusting* for the MAIA-2), which in turn directly influence the observed item responses (see Figure 1 for the MAIA-2), in using the diagonally weighted least square (DWLS) estimation method. Of note, the DWLS is specifically designed for ordinal data, as this is the case for the PAS and the MAIA-2, and is less biased and more accurate than alternative methods (e.g., the maximum likelihood) in estimating the factor loadings (Li, 2016). For the PAS, we fixed the variance of the second-order factor to one, and made the loadings of the two first-order factors equal. Absolute model fit was evaluated with the Root Mean Square Error of Approximation (RMSEA), Standardized Root Mean Square Residual (SRMR), and Comparative Fit Index (CFI) based on common standards (good fit: RMSEA ≤0.05, SRMR ≤0.08, and CFI ≥ 0.95; Hu and Bentler, 1999; Marsh et al., 2004).

#### Reliability

##### Internal Consistency

Reliability of the PAS and the MAIA-2 was assessed using the coefficient omega in considering a higher-order model ($\omega_{HO}$) for the two questionnaires. The rationale for using the coefficient omega, rather than the commonly used Cronbach alpha, is that the latter assumes an essential tau-equivalence model^3^ that appeared to be inappropriate for the PAS and the MAIA-2. As a consequence, the Cronbach alpha can provide misleading reliability estimates (Flora, 2020). In the present paper, values of Cronbach alpha were also reported to provide a comparison with original validation works of the PAS (Cramer et al., 2018) and the MAIA-2 (Mehling et al., 2018).

##### Test–Retest

To ensure that measurement variation reported in our sample is due to replicable differences between participants regardless of time, we performed test–retest reliability analyses. To this end, a subset of participants (*N* = 122) were recalled to complete the PAS and the MAIA-2 questionnaire in a second online testing session [mean (SD) of test–retest interval = 44 (11) days]. Test–retest reliability was quantified by computing the Intraclass Correlation Coefficient (ICC) using the *psych* R package. Briefly, ICC quantifies the extent to which repeated measurements for each individual (within-individual) are statistically similar enough to discriminate between individuals (Aldridge et al., 2017). We used a two-way random effects model for absolute agreement, which corresponds to ICC (2,1) in the Shrout and Fleiss (1979) nomenclature (Shrout and Fleiss, 1979). ICC values less than 0.5, between 0.5 and 0.75, between 0.75 and 0.9, and greater than 0.90 are indicative of poor, moderate, good, and excellent reliability, respectively (Koo and Li, 2016).

#### Construct Validity

We assessed the PAS and the MAIA-2 for convergent and discriminant validity by performing Pearson’s correlations between the two questionnaires and the other three psychological measures (the FMI, the TAS-20, and the BFI-FR). Regarding the convergent validity, we reasoned that if both the PAS and the MAIA-2 measure the construct of body awareness, then individuals felt engaged by information coming from their body should exhibit PAS and MAIA-2 scores that are positively correlated. Since mindfulness has been characterized by enhanced body awareness (Treves et al., 2019), we also expected a positive correlation between PAS and MAIA-2 scores and FMI score. For the discriminant validity, because of the theoretical distinction we make between body awareness and personality, we expected that PAS and MAIA-2 scores do not correlate with BFI-FR subscores. Nevertheless, despite these measurement differences between the BFI-FR and the PAS and MAIA-2 (which measure personality and body awareness respectively), in relation to the negative relationships found between neuroticism and body awareness scores (Trapnell and Campbell, 1999; Ferentzi et al., 2017, 2020), by the mediating role of the emotional regulation difficulties that characterize this personality trait (Gross et al., 1998), we expect to find the same negative correlation in our analyses. Moreover, given perception of bodily signals plays an important role in emotional experience (Critchley and Garfinkel, 2017), alexithymia that characterized individuals having difficulties in identifying their emotions should negatively correlate with postural and interoceptive body awareness.

#### Effect of Non-psychological Factors on Self-Report Measures

We assessed the potential effect on self-reported postural awareness of several “non-psychological” factors, including practice of sport, body-centered practice (e.g., yoga, mindfulness meditation), age, gender, body mass index (BMI) and education level to replicate and extend findings that have been recently published with the Italian version of the PAS (Topino et al., 2020). Based on findings from Topino et al. (2020), we anticipated non-significant statistical results when investigating the effect of gender and age on PAS score (Topino et al., 2020). Interestingly, Bayesian statistical tests have the advantage to provide insight and guide interpretation of non-significant values of *p*, which cannot be interpreted as support for the null hypothesis when using null hypothesis significance testing (Rouder et al., 2009; Wagenmakers et al., 2018). To circumvent this issue, we used both standard statistical tests and Bayesian equivalents. To confirm whether the potential non-significant results reported represent support for the null hypothesis, we calculated the log scale of the Bayes factor [noted log(BF_10_)] that can be easily interpreted such that a negative value indicates support for the null hypothesis, whereas a positive value indicates evidence in favor of the alternative hypothesis [see Supplementary Table S1 for an interpretation scale of log(BF_10_); Jeffreys, 1961]. Standard tests included Mann–Whitney (for the factors practice of sport, body-centered practice, and gender) and Kruskal–Wallis (for the factors education level, age, and BMI that were recoded to categorical variables with more than two classes, see Supplementary Table S2) nonparametric tests. If a significant difference was observed, we computed the effect size (to evaluate the magnitude of the difference) using a measure suited to nonparametric analyses: 95% CI of the rank biserial correlation (Glass, 1966). For the Bayesian analyses, we used the default JASP priors that assume a medium effect size on a Cauchy distribution of 0.707 for independent t-tests, and a *r* scale prior width of 0.5.

## Results

### Socio-Demographic Characteristics

A total of 434 respondents completed the study. Of these, 113 (26%) had incomplete data, and 13 (3%) reported aberrant values for two non-psychological factors of interest (weight < 30 kg or > 200 kg, height < 100 cm or > 230 cm). Thus, these 126 respondents were excluded from the final study sample. The 308 remaining subjects (mean age: 35.22 ± 11.75 years; 189 females—61.40%) were included in the final analyses. This sample was used to compute socio-demographic statistics (Supplementary Table S2), to assess reliability, convergent and discriminant validity of the PAS and the MAIA-2 measures, and to investigate potential effects of non-psychological factors. Subsequently, this sample was randomly split into two subsamples. The first subsample was used for EFA and consisted of 154 subjects (mean age: 36 ± 12 years; 96 females—62%). The second subsample was used for CFA consisted of the remaining 154 subjects (mean age: 35 ± 12 years; 93 females—60%).

### Reliability

#### Internal Consistency

##### Postural Awareness Scale

Overall, internal consistency was satisfactory: for total PAS, the coefficient omega based on a higher-order model ($\omega_{ho}$, see Method section for detailed explanation) was 0.70; for the subscales PAS-EwPA and PAS-NfA, Cronbach alphas were 0.82 and 0.77, respectively (Table 1).

**Table 1:** Descriptive statistics for the Postural Awareness Scale (PAS) and the Multidimensional Assessment of Interoceptive Awareness (MAIA-2) questionnaire in the total sample (*N* = 308).

	M	SD	[Min—Max]	α	$\omega_{ho}$	Range of item-scale correlations^#^
**PAS**						
Total score	45.08 41.2^干^	12.60 10.90^干^	[12–84]	0.85 0.80^干^	0.70	-
Subscale *Familiarity with postural awareness*	22.59 22.20^干^	7.30 6.80^干^	[6–42]	0.82 0.81^干^	0.82	[0.39–0.77]
Subscale *Need for attention regulation with postural awareness*	22.49 19.10^干^	7.15 6.80^干^	[6–42]	0.77 0.77^干^	0.77	[0.44–0.69]
**MAIA-2**						
Total score	23.80	5.11	[9.58–35.93]	0.90 0.74^§^	0.79	-
Subscale *Noticing*	3.44 3.34^§^	1 0.90^§^	[0–5]	0.77 0.64^§^	0.76	[0.64–0.75]
Subscale *Not-distracting*	2.38 2.06^§^	0.84 0.80^§^	[0–4.67]	0.71 0.74^§^	0.57	[0.18–0.39]
Subscale *Not-worrying*	3.10 2.52^§^	0.98 0.85^§^	[0–5]	0.84 0.67^§^	0.84	[−0.08–0.03]
Subscale *Attention regulation*	2.88 2.84^§^	1.04 0.86^§^	[0–5]	0.89 0.83^§^	0.89	[0.75–0.83]
Subscale *Emotional awareness*	3.51 3.44^§^	1.09 0.96^§^	[0–5]	0.85 0.79^§^	0.86	[0.66–0.77]
Subscale *Self-regulation*	2.84 2.78^§^	1.15 1.01^§^	[0–5]	0.85 0.79^§^	0.85	[0.72–0.81]
**MAIA-2**						
Subscale *Body listening*	2.34 2.20^§^	1.18 1.17^§^	[0–5]	0.77 0.80^§^	0.77	[0.72–0.81]
Subscale *Trusting*	3.30 3.37^§^	1.20 1.11^§^	[0–5]	0.84 0.83^§^	0.83	[0.53–0.67]

PAS, Postural Awareness Scale; MAIA-2, Multidimensional Assessment of Interoceptive Awareness (version 2); M, mean; SD, standard deviation; Min, minimum value; Max, maximum value; α, Cronbach alpha; and $\omega_{ho}$, coefficient omega based on a higher-order model.

# Correlations are intended to be descriptive and are not corrected for multiple comparisons.

干 Reference values extracted from the original version of the PAS (Cramer et al., 2018).

§ Reference values extracted from the original version of the MAIA-2 (Mehling et al., 2018).

##### Multidimensional Assessment of Interoceptive Awareness-2

For total MAIA-2, internal consistency was satisfactory: $\omega_{ho}$= 0.79. Cronbach alphas for the eight subscales ranged from 0.71 (MAIA-2-ND) to 0.89 (MAIA-2-AR; Table 1).

#### Test–Retest

##### Postural Awareness Scale

We found evidence that the PAS total score has good reliability over time (ICC = 0.76). The two subscales of the PAS showed moderate reliability with ICCs equal to 0.69 and 0.71 for the subscales PAS-EwPA and PAS-NfA, respectively (Table 2).

**Table 2:** Intraclass correlation coefficients that inform about reliability over time at the individual level for the PAS and the MAIA-2.

	ICC	95% CI
**PAS**		
Total score	0.76	0.69–0.82
Subscale *familiarity with postural awareness*	0.69	0.61–0.76
Subscale *need for attention regulation with postural awareness*	0.71	0.63–0.78
**MAIA-2**		
Total score	0.81	0.75–0.85
Subscale *noticing*	0.69	0.60–0.76
Subscale *not-distracting*	0.66	0.56–0.73
Subscale *not-worrying*	0.72	0.64–0.78
Subscale *attention regulation*	0.63	0.53–0.71
Subscale *emotional awareness*	0.74	0.67–0.80
Subscale *self-regulation*	0.74	0.67–0.80
Subscale *body listening*	0.73	0.65–0.79
Subscale *trusting*	0.82	0.77–0.87

PAS, Postural Awareness Scale; MAIA-2, Multidimensional Assessment of Interoceptive Awareness (version 2); ICC, Intraclass Correlation Coefficient; and 95% CI, 95% confident interval for the Intraclass Correlation Coefficient.

##### Multidimensional Assessment of Interoceptive Awareness-2

We found evidence that the MAIA-2 total score has good reliability over time (ICC = 0.81). Such a good reliability was also observed for the dimension Trusting (ICC = 0.82). Other subscales of the MAIA-2 showed moderate reliability over time, including ICCs that ranged from 0.63 (MAIA-2-AR) to 0.74 (MAIA-2-EA and MAIA-2-SR; Table 2).

### Factor Structure

#### Postural Awareness Scale

##### Exploratory Factor Analysis

A two-factor structure was suggested with the Horn’s Parallel Analysis (Supplementary Figure S1A), explaining 42% of the total variance. The first factor (EwPA) consisted of six items (items 6, 7, 8, 9, 10 and 11) that accounted for 26% of the total variance. The second factor (NfA) was made up of six items (items 1, 2, 3, 4, 5 and 12) that accounted for 16% of the total variance.

##### Confirmatory Factor Analysis

**Figure 2:**
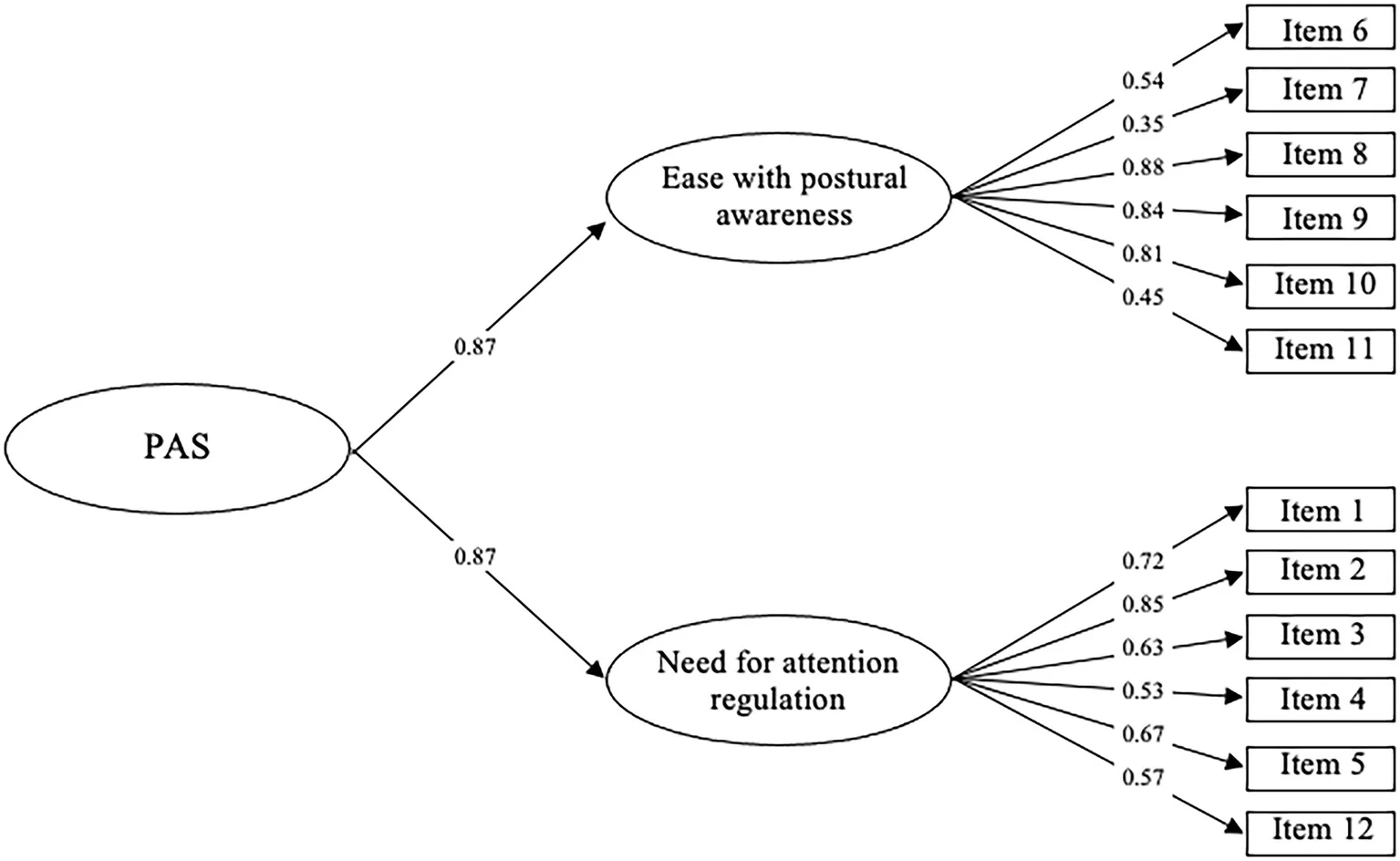
Simplified illustration of the PAS factor model. Values presented represent the standardized regression coefficients.

The higher-order model yielded a good model fit: RMSEA = 0.043 (90% CI: [0–0.070]), SRMR = 0.062, CFI = 0.996. First-order factor loadings range from 0.35 (item 7) to 0.88 (item 8) for the factor *Ease/familiarity with postural awareness*, and from 0.53 (item 4) to 0.85 (item 2) for the factor *Need for attention regulation with postural awareness* (Figure 2). To provide a comparison with the previously published Italian validation of the PAS (Topino et al., 2020), we also report values fit indexes when using Maximum Likelihood (ML) estimation method: RMSEA = 0.055 (90% CI: [X–X]), SRMR = 0.057 CFI = 0.960.

#### Multidimensional Assessment of Interoceptive Awareness-2

##### Exploratory Factor Analysis

Horn’s Parallel Analysis suggested that a six-factor model would be optimal given available data (Supplementary Figure S1B), explaining 55% of the total variance. The six first-order factors accounted for from 6% to 13% of the total variance. It should be noted that an eight-factor model, which is the factor structure originally proposed for the MAIA-2 (Mehling et al., 2018), increased to 60% the proportion of total variance that is explained.

##### Confirmatory Factor Analysis

The higher-order model including a second-order factor on top of six first-order factors, as results of EFA suggested, showed mixed evidence for an acceptable model fit: RMSEA = 0.111 (90% CI: [0.105–0.117]), SRMR = 0.104, CFI = 0.950. When considering the eight-factor model that was originally proposed for the MAIA-2 (Mehling et al., 2018), the model fit increased slightly: RMSEA = 0.106 (90% CI: [0.100–0.112]), SRMR = 0.102, CFI = 0.955. Of note, we also computed values fit indexes when using Maximum Likelihood (ML) estimation method to provide a direct comparison with original validation work of the MAIA-2 (Mehling et al., 2018): RMSEA = 0.075 (90% CI: [0.064–0.078]), SRMR = 0.102, CFI = 0.813. Finally, because it was recently suggested that the first-order factors *Not-distracting* and *Not-worrying* could be independent from the second-order factor *Interoceptive awareness* (Ferentzi et al., 2020), we tested a third higher-order model (Figure 1) in which the first-order factors *Not-distracting* and *Not-worrying* were excluded, and we removed responses to items related to these two factors from the dataset. This latter model yielded the best model fit: RMSEA = 0.079 (90% CI: [0.069–0.088]), SRMR = 0.076, CFI = 0.986. According to this model, first-order factor loadings range from 0.65 (factor *Noticing*, item 1) to 0.97 (factor *Trusting*, item 37; Figure 1).

### Construct Validity

Correlation matrix showed significant correlations with different measures used for the analysis of construct validity of the PAS and the MAIA-2 (Table 3 includes correlation coefficients between total scores, and Supplementary Table S3 includes correlation matrix between all subscales). Descriptive statistics of these measures, including the BFI-FR, the FMI and the TAS-20 questionnaires in the total sample (*N* = 308) are summarized in Supplementary Table S4.

**Table 3:** Pearson’s correlations of the total scores of measures used to assess construct validity.

	1	2	3	4	5	6	7	8	9
1—PAS	1	0.60^**^	0.54^**^	−0.35^**^	0.20^**^	0.23^**^	0.26^**^	−0.29^**^	0.21^**^
2—MAIA-2		1	0.64^**^	−0.50^**^	0.19^*^	0.22^**^	0.28^**^	−0.28^**^	0.25^**^
3—FMI			1	−0.47^**^	0.22^**^	0.34^**^	0.26^**^	−0.58^**^	0.21^**^
4—TAS-20				1	−0.25^**^	−0.25^**^	−0.24^**^	−0.31^**^	−0.24^**^
5—BFI-E					1	0.04	0.23^**^	−0.15^*^	0.25^**^
6—BFI-A						1	0.22^**^	−0.34^**^	0.06
7—BFI-C							1	−0.24^**^	0.05
8—BFI-N								1	−0.02
9—BFI-O									1

* Correlation is significant at the 0.05 level.

** Correlation is significant at the 0.001 level.

Correlations are intended to be descriptive and are not corrected for multiple comparisons. PAS, Postural Awareness Scale; MAIA-2, Multidimensional Assessment of Interoceptive Awareness (version 2); FMI, Freiburg Mindfulness Inventory; TAS20, 20-item Toronto Alexithymia Scale; BFI-E: “Extraversion”; BFI-A: “Agreeableness”; BFI-C: “Conscientiousness,” BFI-N: “Neuroticism”; and BFI-O: “Openness to experience.”

#### Postural Awareness Scale

Both subscales scores were positively, strongly and significantly correlated with the total score (PAS-EwPA, *r* = 0.88, *p* < 0.001 and PAS-NfA *r* = 0.87, *p* < 0.001). The two subscales scores were also significantly intercorrelated (*r* = 0.52, *p* < 0.001).

##### Convergent Validity

Overall, the pattern of correlations (direction and significance) with the different measures was similar for the PAS total score and its two subscales. Of note, the measure of MAIA-2-NW was an exception in that it only correlated with the PAS-EwPA subscale (*r* = − 0.14, *p* < 0.05). Specifically, we observed strong positive correlation between the PAS and the MAIA-2 total scores (*r* = 0.60, *p* < 0.001). Positive correlations were found between the PAS total score and the MAIA-2’s subscales, ranging from r = 0.21, *p* < 0.001 (MAIA-2-ND) to *r* = 0.54, *p* < 0.05 (MAIA-2-AR; see Supplementary Table S3). We also observed positive correlations between all PAS scores (total and subscales) and the FMI total score (*r* = 0.47, *p* < 0.001 for PAS-EwFA and PAS-NfA; *r* = 0.54, *p* < 0.001 for PAS total score). Similar positive correlations were found with the two FMI subscales (see Supplementary Table S3). All PAS scores were moderately and positively correlated with the BFI-E, the BFI- A, the BFI-C, and the BFI-O (ranging from *r* = 0.14, *p* < 0.05 to *r* = 0.26, *p* < 0.001; see Supplementary Table S3).

##### Discriminant Validity

Negative correlations were found with all TAS scores, ranging from *r* = − 0.16, *p* < 0.001 (PAS-EwPA and TAS-DIF and TAS-DVE) to *r* = − 0.40, *p* < 0.001 (PAS-NfA and TAS-DIF). PAS total and subscales were also negatively, but moderately, correlated with the BFI-N (the weakest correlation: *r* = − 0.14, *p* < 0.05 for PAS-EwFA; Supplementary Table S3).

#### Multidimensional Assessment of Interoceptive Awareness-2

##### Convergent Validity

All MAIA-2 scores (total and subscales) showed positive correlation with the FMI total score (*r* = 0.64, *p* < 0.001 for the MAIA-2 total score, and from *r* = 0.16, *p* < 0.001 (MAIA-2-ND) to *r* = 0.58, *p* < 0.001 (MAIA-2-SR) for MAIA-2 subscales; see Supplementary Table S3).

##### Discriminant Validity

Negative correlation has been found between the MAIA-2 total score and the total score of the TAS-20 (*r* = −0.50, *p* < 0.001), as well as the dimension Neuroticism of the BFI-FR (*r* = −0.28, *p* < 0.001). Almost all subscale scores of the MAIA-2 (except one: Not-worrying) were significantly negatively correlated with the TAS total score, ranging from *r* = −0.27, *p* < 0.001 (MAIA-2-NW) to *r* = −0.42, *p* < 0.001 (MAIA-2-T). Regarding the dimension Neuroticism of the BFI-FR, it was negatively correlated with four MAIA-2 subscales, ranging from *r* = −0.24, *p* < 0.001 (MAIA-2-NW) to *r* = −0.44, *p* < 0.001 (MAIA-2-T).

### Effect of Non-psychological Factors on Self-Report Measures

Supplementary Table S5 summarizes statistics that inform the effects of categorical non-psychological factors (sport practice, body-centered activity, and gender) on the self-report measure of interoceptive (MAIA-2) and postural (PAS) body awareness.

#### Practice of Sport

Individuals who reported practice of sport showed significantly higher score for the dimensions PAS-NfA, MAIA-2-AR and MAIA-2-T, compared to individuals who did not. For the dimension PAS-EwPA, individuals who reported practice of sport tend to have a higher score than those who did not. There was no significant effect of sport practice on other subscales of the MAIA-2.

#### Body-Centered Activity

**Figure 3:**
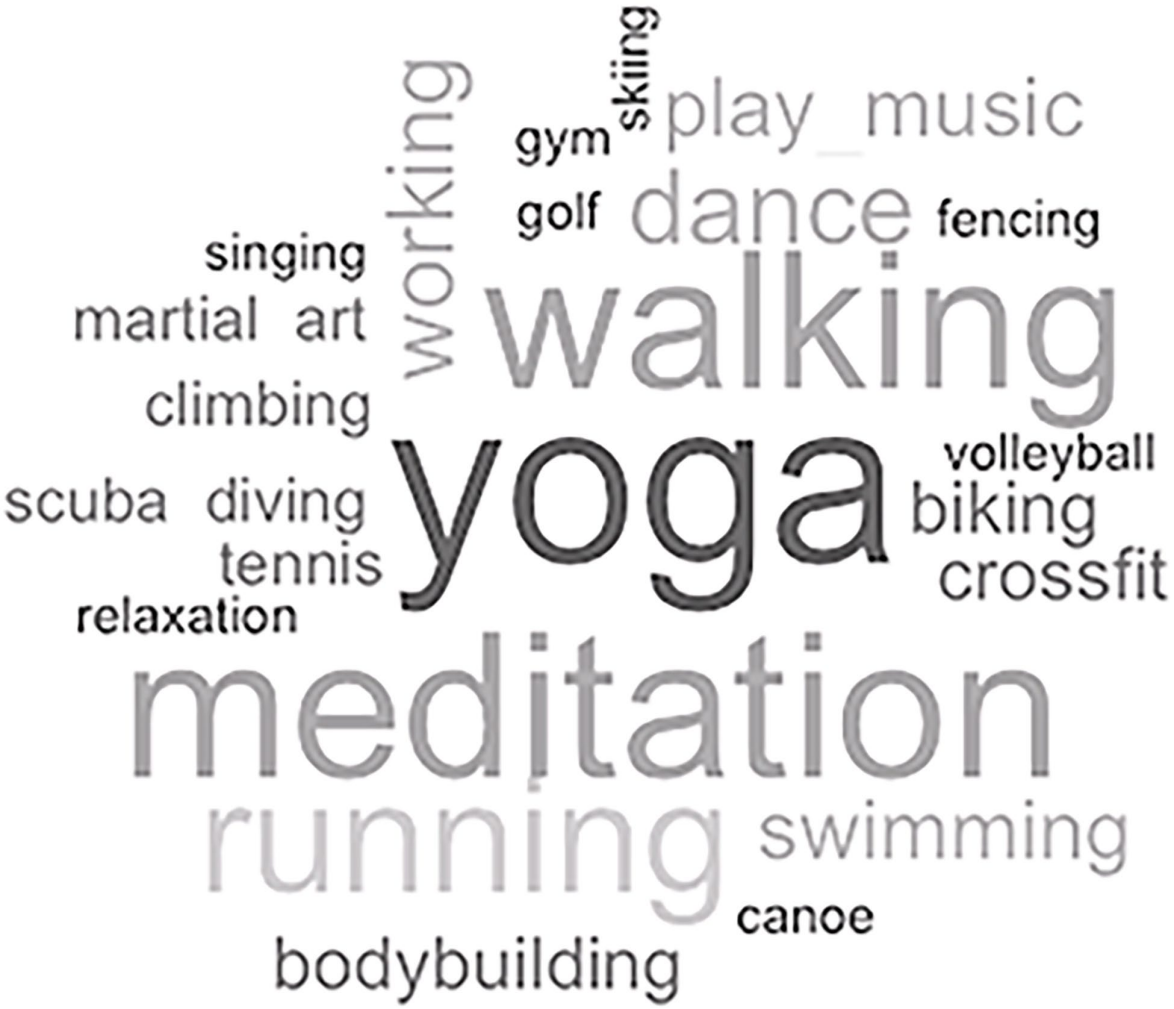
Word cloud of body-centered activities that were reported in our sample based on their relative frequency. The bigger the word, the greater the frequency influences. The figure is a representation of words that have been entered at least more than twice (minimum frequency = 2).

Figure 3 describes the body-centered activities that were reported in our sample. Individuals who reported practice of a regular body-centered activity showed significantly higher scores for all the dimensions of the PAS and the MAIA-2, except for the dimensions MAIA-2-ND and MAIA-2-NW, compared to individuals who did not.

#### Gender

We did not find any effect of the gender on self-reported postural body awareness. By contrast, regarding the interoceptive body awareness, we found that scores for the dimensions MAIA-2-N, MAIA-2-EA, and MAIA-2-BL were significantly higher in females than in males. Furthermore, the score for the dimension MAIA-2-T was significantly higher in males than in females.

#### Age

None of the dimensions of the PAS and the MAIA-2 did correlate with age of participants, with log(BF_10_) ranging from −1.30 (MAIA-2-N) to −2.63 (MAIA-2-BL) suggesting extreme evidence for the null hypothesis. To provide a direct comparison with results from Topino et al. (2020), we also tested the effect of age when transforming as a categorical variable in using Topino’s criteria (Topino et al., 2020) on the PAS subscales. We did not find any effect of age classes on the two dimensions of the PAS-EwPA: log(BF_10_) = −2.51, suggesting extreme evidence for the null hypothesis; PAS-NfA: log(BF_10_) = −3.92, suggesting extreme evidence for the null hypothesis.

#### Education Level

We did not find any effect of the education level on self-reported postural body awareness, with log(BF_10_) ranging from −2.86 (PAS-EwPA) to −3.34 (PAS-NfA) suggesting extreme evidence for the null hypothesis. Regarding the interoceptive awareness, there was no significant effect of education level on the eight subscales of the MAIA-2, with log(BF_10_) ranging from −3.20 (MAIA-2-T), which suggests extreme evidence for the null hypothesis, to 0.45 (MAIA-2-EA) suggesting no evidence for the alternative hypothesis.

#### Body Mass Index

All the dimensions of the PAS and most of the dimensions of the MAIA-2 (except the dimension MAIA-2-T) did not correlate with BMI of participants, with log(BF_10_) ranging from −1.12 (MAIA-2-BL) to −2.63 (PAS-EwPA), suggesting strong to extreme evidence for the null hypothesis. The dimension MAIA-2-T showed significant negative correlation with the BMI of participants (*r* = −0.21, *p* < 0.001). To provide a direct comparison with results from Topino et al. (2020), we also tested the effect of BMI when transforming as a categorical variable in using Topino’s criteria (Topino et al., 2020) on the PAS subscales. We did not find any effect of BMI classes on the two dimensions of the PAS-EWPA: log(BF_10_) = −1.59, suggesting very strong evidence for the null hypothesis; PAS-NfA: log(BF_10_) = −2.78, suggesting extreme evidence for the null hypothesis.

## Discussion

### General Discussion

The aim of this study was to evaluate psychometric properties and validate in French the PAS, a recently developed questionnaire to assess postural body awareness (Cramer et al., 2018), and the MAIA-2, which is the latest version of a popular questionnaire assessing interoception (the interoceptive component of body awareness; Mehling et al., 2018). Our data, collected in a non-clinical adult sample, showed that the French version of the PAS and the MAIA-2 have both good construct validity and good internal consistency, as well as a good reliability over time. First, regarding the construct validity of the two questionnaires, significant positive correlations were found with the dispositional trait mindfulness, which is characterized by enhanced body awareness (Treves et al., 2019). Our finding is consistent with previously published applications of the MAIA (Mehling et al., 2012; Bornemann et al., 2015; Hanley et al., 2017; Verdonk et al., 2021) and the PAS (Cramer et al., 2018; Topino et al., 2020). On the other hand, scores of the PAS and the MAIA-2 showed negative correlation with alexithymia (inability to identify and describe emotions in the self), which is a psychological construct that is theoretically and empirically in opposition to body awareness (Herbert et al., 2011; Murphy et al., 2018a,b; Zamariola et al., 2018; Topino et al., 2020). This result suggests the idea that individuals with alexithymia may have a disrupted processing of bodily signals, which could ultimately lead to impairments in emotional awareness since the ability to feel bodily sensations is thought to be a central antecedent of the conscious experience of emotions (Zamariola et al., 2018). This is in line with studies that have shown a general failure of interoception, as measured by heartbeat perception task, in alexithymia (Brewer et al., 2016). Regarding the dimension *Neuroticism* of the BFI-FR, our analyses showed significantly negative correlation with the interoceptive dimensions of *Attention Regulation*, *Self-regulation*, *Trusting*, and *Not-worrying*. This finding is in line with the work from Pearson and Pfeifer (2020) but contrasts with results from Ferentzi et al. (Ferentzi et al., 2020; Pearson and Pfeifer, 2020). Neuroticism is considered as an individual’s tendency to worry and be anxious, as well as to overreact to negative affect (Costa and McCrae, 1992). Previous studies have reported that higher neuroticism individuals have a diminished ability to regulate emotion regulation, specifically a diminished capacity to downregulate negative emotions (Harenski et al., 2009; Yang et al., 2020). Our finding suggests that difficulty experienced by individuals with high neuroticism in regulating their emotion could partly result from inability to actively pay attention to their body sensations, which are proposed to shape emotional experience (Critchley and Nagai, 2012; Critchley and Garfinkel, 2017). Taken together, our results suggest that the psychological construct of body awareness, i.e., the ability to feel engaged by information coming from the body, might potentially play a mediator role in the relationship between personality traits, such as neuroticism and alexithymia, and emotion dysregulation (Harenski et al., 2009; Yang et al., 2020; Preece et al., 2021). Specifically, we suggest that low level of emotion regulation characterizing people with high level of neuroticism, on one hand, and inability of people with alexithymia to identify, describe and thus regulate their emotions on the other hand, may be responsible for the negative correlation reported between these two factors and body awareness scores. This hypothesis needs to be tested in further studies by using mediation analyses to reveal potential role of body awareness in the transmission of (causal) effect of personality traits to emotion dysregulation (Baron and Kenny, 1986; Agler and De Boeck, 2017). Regarding other personality dimensions assessed with the BFI-FR, we reported a moderate positive correlation between scores of the PAS and the MAIA-2 and the dimensions of *Conscientiousness*, *Extraversion* and *Openness*. These results are consistent with the findings from Ferentzi et al. (2017, 2020), and are in line with our expectations, especially for the dimension of Openness to experience, which is characterized by the tendency to engage in body-related activities (Wilson and Dishman, 2015; Sutin et al., 2016), as well as for the dimension of Conscientiousness characterized by self-control abilities that require good body awareness (Costa and McCrae, 1992). Regarding the association between Extraversion and scores on the PAS and MAIA-2, our results are in line with findings from Baer et al. (2004) suggesting that this personality dimension is related to the ability of extraverted individuals to describe their internal experiences. They point out that the body awareness of people with high levels of extraversion could be explained by their ability to put words to their experiences through speech and social interaction (Baer et al., 2004). Finally, we observed that self-reported postural and interoceptive body awareness strongly and positively correlate to each other, thus suggesting that proprioception and interoception refer to two components of a homogeneous, unified psychological construct of body awareness. Interestingly, recent neuroimaging studies also accounted for this hypothesis by highlighting that some of the brain areas involved in the processing of interoceptive and proprioceptive signals, notably the parietal cortex, could overlap (García-Cordero et al., 2017; Salvato et al., 2020). It has also been shown that redundancy and complementarity characterize signals originating from within the body, and such features appear to be functionally relevant for cardiac interoception (Khalsa et al., 2009), as well as for postural response in stressful situations (Volchan et al., 2017).

Factor analyses showed that the French version of the PAS has the same underlying two-factor structure as previously published versions (Cramer et al., 2018; Topino et al., 2020). The first factor regards the ability to have a high postural awareness in a natural and effortless way (Ease/familiarity with postural awareness), and the second refers to the need for high efforts to be aware of their own posture (Need for attention regulation with postural awareness). Regarding the French version of the MAIA-2, results from EFA suggested a model in which the optimal number of factors is limited to six. This model differs from the eight-factor model that has been proposed with the first version of the MAIA (MAIA-1; Mehling et al., 2012). Of note, the recent development of a modified version of the MAIA (MAIA-2) aimed to improve its psychometrics by adding new items to the *Not-worrying* and *Not-distracting* subscales, which have been reported to be of limited internal consistency reliability in numerous applications (Mehling et al., 2018). We observed that *Not-worrying* and *Not-distracting* scores are only weakly correlated with MAIA-2 total score, in line with the recent work from Ferentzi et al. (2020). They suggested that *Not-worrying* and *Not-distracting* subscales could be unrelated to the common general factor of interoceptive body awareness. Based on this hypothesis, we performed additional CFA on a subset of the original dataset, in which responses to items related to *Not-worrying* and *Not-distracting* factors were removed, and we found the best model fit with a six-factor model including factors of *Noticing*, *Attention regulation*, *Emotional Awareness*, *Self-regulation*, *Body listening*, and *Trusting*. Our findings, which need to be confirmed in a larger French-speaking sample, contribute to the call for a reconsideration of the MAIA structure, in particular the relevance of keeping items that are related to *Not-worrying* and *Not-distracting* factors. Nonetheless, the reader should bear in mind that differences in model fit between the six-factor and the eight-factor models remain relatively small, thus supporting the 37-item MAIA-2 as an appropriate instrument for interoception research to assess subjective body awareness.

In our work, we also investigated the effect of “non-psychological” factors on the PAS and the MAIA-2 scores. In line with findings of the Italian version of the PAS (Topino et al., 2020), we found that practices of sport and body-centered activity are associated with higher self-reported postural awareness. Contrary to results from Topino et al. (2020), we did not observe any significant relationship between BMI and PAS score. For the MAIA-2 questionnaire, we also found that practices of sport and body-centered activity are associated with higher self-reported interoceptive body awareness. Furthermore, we observed a significant effect of gender on the dimensions *Noticing*, *Emotional Awareness*, *Body listening*, and *Trusting*, which is consistent with findings from interoception literature (Grabauskaitė et al., 2017).

### Limitations, Constraints on Generality, and Perspectives

This study has some limitations that might need to be addressed in future research. First, this study was not pre-registered. The assumptions and analyses made in this study were derived from the original design studies of the PAS (Cramer et al., 2018) and MAIA-2 (Mehling et al., 2018), as well as validation articles of these scales available in other languages (Topino et al., 2020 for the PAS; Ferentzi et al., 2020 for the MAIA). Secondly, concerning the conduct of the study, a relatively small number of self-report measures were collected to test construct validity of the PAS and the MAIA-2. This results from the limited collection of questionnaires used in interoception research that are currently validated in the French-speaking population. Regarding the data collected, it should be noted that no control measures were carried out on psychiatric symptoms that could affect self-reported body awareness, although people with neurological or psychiatric illnesses requiring psychotherapeutic and/or drug treatment were asked not to respond to our questionnaires. Some data from people with current or ongoing psychopathological symptoms that affect their level of body awareness could therefore have been considered and contributed to influencing the scores we find for these two questionnaires. Similarly, no individual-level measures were proposed to monitor participants’ actual ability to speak and understand French and the questionnaire items correctly. We ensured that the instructions and items of the PAS and MAIA-2 questionnaires were unanimously understood by conducting a pilot test on a small sample of French people. We also asked participants not to respond to our study if they did not master the language. Our sample included a relatively large proportion of participants with an education level higher than 2 years of university courses (*n* = 244—79.22%), and hence differs from the French general population in which the proportion of individuals reporting more than 2 years of university courses is between 28.6% and 36.1%. Of note, our data showed that education level does not affect self-reported interoception and postural awareness. Participation in the second phase of the study, i.e., the retest, was relatively limited with only 122 of the 306 participants (40%) completing the PAS and MAIA-2 a second time. There are also some limitations inherent in the self-report psychological scales, among which social desirability and response bias, but in the field of body awareness self-report questionnaire seem to be one of the most relevant tools. Indeed, like patient reported outcome—PRO—used in chronic pathologies (such as chronic arthritis or irritable bowel syndrome) to assess how well patients respond to treatment from the patients’ perspective, assessment of body awareness has to be patient/subject centered and this is made possible by self-report measures. Furthermore, one could argue that self-report questionnaires are only one of the well-established methods of capturing individual differences in psychology. Objective measures, including behavioral tests and physiological signals, are also of particular interest to investigate inter-subject variability in the process of sensing signals coming from the body. Behavioral tests enable objective measure of body awareness (e.g., heartbeat perception task for cardiac interoception; Brener and Ring, 2016), but their features that make them robust in an experimental sense make behavioral tests unreliable in a psychometric sense (Hedge et al., 2018). In addition, behavioral tests and self-report questionnaires inform about two dissociable dimensions of body awareness, the body awareness accuracy (i.e., performance on bodily signal detection tasks) and body awareness sensibility (i.e., degree to which individuals feel engaged by bodily signals) respectively, according to the model of (cardiac interoceptive) body awareness proposed by Garfinkel et al. (2015). Regarding physiological signals associated with body awareness, the Heartbeat Evoked Potential (HEP), which refers to evoked changes in brain activity (measured using magnetoencephalography, electroencephalography, or intracranial neural recordings) that occurs after a heartbeat, has been proposed as a neurophysiological marker of interoception (Coll et al., 2021). It should be noted that Verdonk et al. (2021) have recently shown that the HEP amplitude is not associated with the self-reported interoceptive awareness, as measured with the MAIA-1. Regarding the postural component of body awareness, we suggest that the postural signal could be a candidate physiological biomarker to assess construct validity of the PAS. Future studies are encouraged to investigate the relationship the self-reported postural body awareness, as measured with the PAS, could have with the postural signal recorded during standing posture.

## Data Availability Statement

All requests for raw and analyzed data should be addressed to dcssa-paris@sante.defense.gouv.fr, because they will be reviewed by our legal department (French Military Health Service) to verify whether the request is subject to any confidentiality constraints. Requests regarding materials, including programming code, should be addressed to the corresponding author (ChV).

## Ethics Statement

The study has been reviewed and approved by the H.I.A. Saint Anne Ethics Committee (0011873-2021-02). The participants provided their written informed consent to participate in this study.

## Author Contributions

LD, AD, and ChV equally contributed to the work, including conceptualization of the research question, design of the methodology, execution of the study, data analysis, and writing of the paper. CB, BR, ES, CoV, and MT actively took part in the process of cross-cultural translation of the two questionnaires. All authors approved the submitted version of the article.

## Author Disclaimer

The opinions or assertions expressed herein are the private views of the authors and are not to be considered as official or as reflecting the views of the French Military Health Service.

## Conflict of Interest

ES is the sole proprietor of Traduction Édition Scientifique.

The remaining authors declare that the research was conducted in the absence of any commercial or financial relationships that could be construed as a potential conflict of interest.

## Correction Note

A correction has been made to this article. Details can be found at: 10.3389/fpsyg.2026.1989051.

## Publisher’s Note

All claims expressed in this article are solely those of the authors and do not necessarily represent those of their affiliated organizations, or those of the publisher, the editors and the reviewers. Any product that may be evaluated in this article, or claim that may be made by its manufacturer, is not guaranteed or endorsed by the publisher.

## Footnotes

^1^ https://www.limesurvey.org

^2^ https://jasp-stats.org/

^3^A tau-equivalence model includes a single factor (i.e., the model is unidimensional) that shows equal factor loadings across all items (Flora, 2020).
