# Correction: Self-Reported Body Awareness: Validation of the Postural Awareness Scale and the Multidimensional Assessment of Interoceptive Awareness (Version 2) in a Non-clinical Adult French-Speaking Sample

**DOI:** 10.3389/fpsyg.2026.1989051

**Published:** 2026-10-05

**Authors:** Lucie Da Costa Silva, Célia Belrose, Marion Trousselard, Blake Rea, Elaine Seery, Constance Verdonk, Anaïs M. Duffaud, Charles Verdonk

**Affiliations:** 1 Department of Neurosciences and Cognitive Sciences, Unit of Neurophysiology of Stress, French Armed Forces Biomedical Research Institute, Brétigny-sur-Orge, France; 2 Traduction Édition Scientifique, Toulouse, France; 3 Department of Cardiac Surgery and Cardiology, Bichat-Hospital, Paris, France

**Keywords:** body awareness, proprioception, interoception, PAS, MAIA-2

In the published article, there was an error in the description of the reverse-scored items of the Multidimensional Assessment of Interoceptive Awareness, Version 2 (MAIA-2).

A correction has been made to **Materials and Methods**, *Multidimensional Assessment of Interoceptive Awareness (Version 2)*. The sentence previously stated:

“For each of the eight subscales, the score was counted by averaging the scores of items belonging to the subscale (items 5, 6, 7, 8 and 9 were reversed).”

The corrected sentence appears below:

“For each of the eight subscales, the score was counted by averaging the scores of items belonging to the subscale (items 5, 6, 7, 8, 9, 10, 11, 12, and 15 were reverse scored).”

The original article has been updated.

