# Supplementary material for: Self-Reported Body Awareness: Validation of the Postural Awareness Scale and the Multidimensional Assessment of Interoceptive Awareness (Version 2) in a Non-clinical Adult French-Speaking Sample
Source: Front Psychol. 2022 Jul 26;13:946271. doi: 10.3389/fpsyg.2022.946271 (PMC9362853; doi:10.3389/fpsyg.2022.946271)
Supplement: Supplementary file 1 [file Data_Sheet_1.PDF]

Supplementary

Table S1 - A descriptive and approximate classification scheme for the interpretation of the log scale of Bayes factor BF_{10} (adapted from Jeffreys, 1961)

Log (BF_{10})	Interpretation	Symbol
> 2	extreme evidence for H_1	H_1^{****}
[1.48 ; 2]	very strong evidence for H_1	H_1^{***}
[1 ; 1.48]	strong evidence for H_1	H_1^{**}
[0.48 ; 1]	moderate evidence for H_1	H_1^{*}
[0 ; 0.48]	anecdotal evidence for H_1	ns
0	no evidence	ns
[-0.48 ; 0]	anecdotal evidence for H_0	ns
[-1 ; -0.48]	moderate evidence for H_0	H_0^{*}
[-1.48 ; -1]	strong evidence for H_0	H_0^{**}
[-2 ; -1.48]	very strong evidence for H_0	H_0^{***}
< -2	extreme evidence for H_0	H_0^{****}

Growing evidence in favor of H_1

Growing evidence in favor of H_0

log(BF_{10}): log scale of Bayes factor BF_{10}; H_1: alternative hypothesis; ns: non-significant; H_0: null hypothesis

Table S2 - Summary of socio-demographic data

	N	%
**Gender**		
Male	119	61.4
Female	189	38.6
**Body Mass Index** (M ± SD : 23.93 ± 4.18)		
Obesity (class 2 & 3) : BMI ≥ 35	7	2.3
Obseity (class 1) : BMI [30 – 35[	13	4.2
Overweight : BMI [25 – 30[	76	24.7
Normal weight : BMI [18.5 – 25[	203	65.9
Underweight : BMI < 18.5	9	2.9
**Study degree**		
Middle school diploma	21	6.8
High school diploma	31	10.1
Higher national diploma	33	10.7
University degree	16	5.2
Bachelor degree	90	29.2
Doctoral degree	105	34.1
Other	12	3.9
**Sport practice**		
Yes	208	67.5
No	100	32.5

	N	%
**Sport**		
Gym	2	0.6
Water sports	8	2.6
Football	5	1.6
Cycling and running	84	27.3
Walking and trekking	18	5.8
Bodyweight exercises, yoga, fitness	50	16.2
Dance and skating	3	1.0
Volley	4	1.3
Basket and rugby	2	0.6
Martial arts and combat sports	11	3.6
Other	21	6.8
**Practice of a body-centered activity**		
Yes	146	46.4
No	162	52.6

N: number of participants; %: proportion of participants; M: mean; SD: standard deviation; BMI: Body Mass Index

Table S3 - Pearson's correlations of the measures used to assess construct validity

	1	2	3	4	5	6	7	8	9	10	11	12	13	14	15	16	17	18	19	20	21	22	23	24
1- PAS	1	.87**	.88**	.60**	.45**	.21**	-.06	.54*	.39**	.43**	.45**	.40**	.54**	.53**	.47**	-.35**	-.31**	-.22**	-31**	.20**	.23**	.26**	-.29**	.21**
2 - PAS-EwPA		1	.52**	.56**	.46**	.14*	-.14*	.48**	.44**	.43**	.49**	.30**	.47**	.50**	.39**	-.26**	-.16**	-.16**	-.31**	.19**	.17*	.19**	-.14*	.22**
3 - PAS-NfA			1	.48**	.32**	.23**	.04	.46**	.23**	.33**	.28**	.39**	.47**	.44**	.43**	-.36**	-.40**	-.23**	-.23**	.16**	.22**	.26**	-.37**	.14*
4 - MAIA-2				1	.70**	.29**	.03	.80**	.72**	.77**	.77**	.61**	.64**	.62**	.56**	-.50**	-.38**	-.32**	-.51**	.19*	.22**	.28**	-.28**	.25**
5 - MAIA-2-N					1	.20**	- .22**	.56**	.58**	.42**	.51**	.27**	.38**	.43**	.28**	-.30**	-16**	-.21**	-.37**	.14*	.08	.20**	-.03	.21**
6 - MAIA-2-ND						1	- .21**	.13**	.04	.05	.22**	.13*	.16**	.16*	.14*	-.27**	-.17**	-.23**	-.26**	-.03	.06	-.04	.04	.14*
7 - MAIA-2-NW							1	.04	-.17**	-.10	-.26**	.11	-.07	-.16*	.02	0.2	-.18**	.11	.13*	-.01	.09	.04	-.24**	-.
8 - MAIA-2-AR								1	.52**	.56**	.52**	.41**	.54**	.52**	.47**	-.37**	-.30**	-.21**	-.37**	.13*	.14*	.24**	-.26**	.24**
9 - MAIA-2-EA									1	.54**	.64**	.20**	.35**	.42**	.24**	-.30**	-.12*	-.21**	-.41**	.14*	.14*	.26**	.03	.14*
10 - MAIA-2-SR										1	.64**	.41**	.58**	.55**	.52**	-.37**	-.27**	-.23**	-.39**	.07	.18**	.22**	-.30**	.12**
11 - MAIA-2-BL											1	.28**	.48**	.49**	.40**	-.33**	-.12*	-.25**	-.45**	.16*	.17**	.22**	-.07	.27**
12 - MAIA-2-TR												1	.53**	.44**	.52**	-.42**	-.45**	-.29**	-.26**	.24**	.18**	.17**	.-.44**	-.15**
13 - FMI													1	0.90**	.93**	-.47**	-.39**	-.31**	-.37**	.22**	.34**	.26**	-.58**	.21**
14 - FMI-P														1	.68**	-.40**	-.33**	-.28**	-.37**	.21**	.26**	.31**	-.43**	.18**
15 - FMI-A															1	-.44**	-.40**	-.31**	-.37**	.20**	.34**	.18**	-.61**	.20**
16 - TAS-20																1	.83**	.85**	.73**	-.25**	-.25**	-.24**	-.31**	-.24**
17 - TAS-DIF																	1	.60**	.35**	-.13*	-.19**	-.24**	.46**	-.07
18 - TAS- DVE																		1	0.45**	-.29**	-.20**	-.19**	.22**	-.16**
19 - TAS-EOT																			1	-.17**	-.23**	-.14*	.05	-.37**
20 - BFI-E																				1	.04	.23**	-.15*	.25**
21 - BFI-A																					1	.22**	-.34**	.06
22 - BFI-C																						1	-.24**	.05
23 - BFI-N																							1	-.02
24 - BFI-O																								1

*Correlation is significant at the 0.05 level; ** Correlation is significant at the 0.001 level; PAS: Postural Awareness Scale; PAS EwPA: “Ease/familiarity with postural awareness”; PAS NfA: “Need for attention regulation with postural awareness”; FMI: Freiburg Mindfulness Inventory; FMI-P: “Presence”; FMI-A: “Acceptation”; MAIA-2: Multidimensional Assessment of Interoceptive Awareness (version 2); MAIA-2-N: “Noticing”; MAIA-2-ND: “Not-Distracting”; MAIA-2-NW: “Not-Worrying”; MAIA-2-AR: “Attention Regulation”; MAIA-2-EA: “Emotional Awareness”; MAIA-2-SR: “Self-Regulation”; MAIA-2-BL: “Body Listening”; MAIA-2-TR: “Trusting”. TAS20: 20-item Toronto Alexithymia Scale; TAS-DIF: “Difficulty identifying feelings and distinguishing between feelings and bodily sensations in emotional activation”; TAS-DVE: “Difficulty in the verbal expression of emotions”; TAS-EOT: “Externally oriented thinking”. BFI-E: “Extraversion”; BFI-A: “Agreeableness”; BFI-C: “Conscientiousness”, BFI-N: “Neuroticism”; BFI-O: “Openness to experience”.

Table S4 - Descriptive statistics for the BFI-FR, the FMI and the TAS-20 questionnaires on the total sample (N=308)

	M	SD	[Min - Max]	α	ω	Range of item-scale correlations[#]
**BFI-FR**						
BFI-O	36.84	6.30	[22 - 50]	0.77	-	-
BFI-C	33.19	5.95	[14 - 44]	0.82	-	-
BFI-E	25.83	6.82	[9 - 40]	0.86	-	-
BFI-A	39.18	5.80	[21 - 49]	0.78	-	-
BFI-N	23.14	7.21	[8 - 40]	0.88	-	-
**FMI**						
Presence	16.71	3.59	[6 – 25]	0.80	$\omega_{u\text{-}cat}$ = 0.81	[0.57 – 0.73]
Acceptation	20.23	4.46	[8 – 32]	0.75	$\omega_{u\text{-}cat}$ = 0.78	[0.33 – 0.67]
Total score	36.94	7.39	[15 – 56]	0.86	$\omega_{u\text{-}cat}$ = 0.89	-
**TAS-20**						
Difficulty identifying feelings	16.44	5.44	[7 – 31]	0.77	ω_{ho} = 0.80	[0.11 – 0.72]
Difficulty in the verbal expression of emotions	14.69	5.11	[5 – 25]	0.81	ω_{ho} = 0.80	[0.55 – 0.79]
Externally oriented thinking	17.84	4.72	[8 – 31]	0.59	ω_{ho} = 0.61	[0.17 – 0.54]
Total score	48.97	12.28	[20 – 86]	0.84	ω_{ho} = 0.75	-

BFI-FR: French version of the Big Five Inventory; FMI : Freiburg Mindfulness Inventory; TAS-20 : Toronto Alexithymia Scale; M: mean; SD: standard deviation; Min: minimum value; Max: maximum value; α: Cronbach alpha; ω_{ho}: coefficient omega based on a higher-order model; ω_{u-cat}: categorical omega; [#]Correlations are intended to be descriptive and are not corrected for multiple comparisons

Table S5 - Effects of categorical non-psychological factors (sport practice, body-centered activity, and gender). Standard statistics and Bayesian equivalents inform the effects of factors on psychometric scores.

	**Practice of sport**				**Body-centered activity**				**Gender**			
	Stat	*p*	*BF*	*Int.*	*Stat*	*p*	*BF*	*Int.*	*Stat*	*p*	*BF*	*Int.*
**PAS**												
Total score	8097	<0.01	-	✓ > ✗	8213	<0.001	-	✓ > ✗	10187	0.164	-1.29	-
Subscale *Familiarity with postural awareness*	9016	0.06	-	✓ > ✗	8468	<0.001	-	✓ > ✗	10982	0.73	-1.86	-
Subscale *Need for attention regulation with postural awareness*	7747	<0.001	-	✓ > ✗	9144	<0.001	-	✓ > ✗	9731	0.06	- 0.74	-
**MAIA-2**												
Total score	8641	<0.05	-	✓ > ✗	8784	<0.001	-	✓ > ✗	11941	0.36	-1.53	-
Subscale *Noticing*	9388	0.17	-1.34	-	9815	<0.05	-	✓ > ✗	13485	<0.01	-	♀ > ♂
Subscale *Not-distracting*	11118	0.33	-1.65	-	10661	0.14	-1.19	-	10820	0.58	-1.85	-
Subscale *Not-worrying*	9429	0.18	-1.42	-	12221	0.61	-2.00	-	11011	0.76	-2.06	-
Subscale *Attention regulation*	8225	<0.01	-	✓ > ✗	9397	<0.01	-	✓ > ✗	12029	0.30	-1.68	-

	**Practice of sport**				**Body-centered activity**				**Gender**			
	Stat	*p*	*BF*	*Int.*	*Stat*	*p*	*BF*	*Int.*	*Stat*	*p*	*BF*	*Int.*
Subscale *Emotional awareness*	9786	0.40	-1.92	-	9873	<0.05	-	✓ > ✗	13331	<0.01	-	♀ > ♂
Subscale *Self-regulation*	9245	0.11	-0.91	-	8924	<0.001	-	✓ > ✗	11065	0.81	-2.03	-
Subscale *Body listening*	10057	0.64	-1.91	-	9650	<0.01	-	✓ > ✗	13002	<0.05	-	♀ > ♂
Subscale *Trusting*	7482	<0.001	-	✓ > ✗	9610	<0.01	-	✓ > ✗	9156	<0.01	-	♀ < ♂

PAS: Postural Awareness Scale; MAIA-2: Multidimensional Assessment of Interoceptive Awareness (version 2); *Stat* : statistic ; *p* : p-value ; *BF* : log scale of Bayes factor BF_{10} ; *Int.* : interpretation of betwen-modality comparisons; ✓ : practice of sport or body-centered activity ; ✗ : no sport practice or body-centered activity ; ♀ : female gender ; ♂ : male gender

Figures

Figure S1

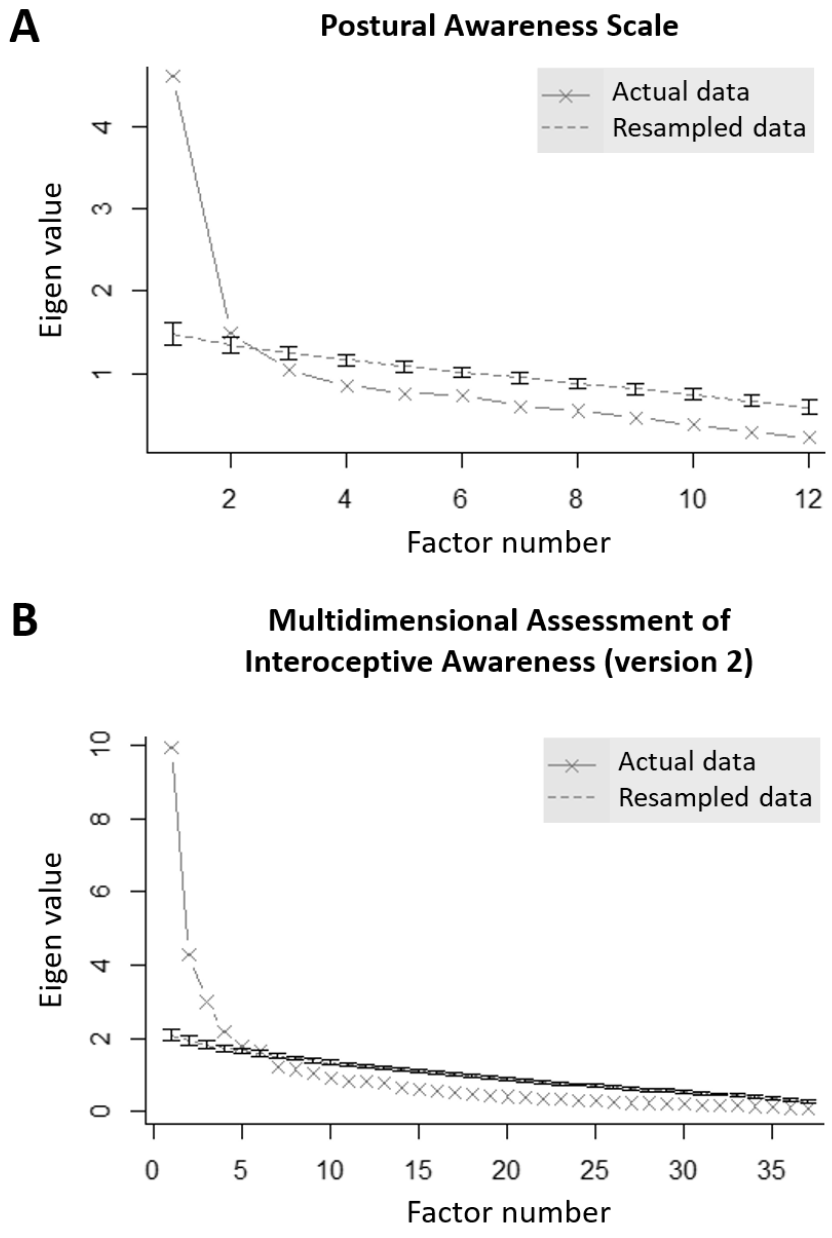

Figure S1 - Scree plot for **(A)** the Postural Awareness Scale and **(B)** the Multidimensional Assessment of Interoceptive Awareness (version 2). The figure includes the 95% confidence interval of eigenvalues obtained after bootstrapping using 1000 replications.

Supplementary Reference

Jeffreys, H. (1961). *Theory of probability* (Clarendon Press Ed.).

Postural Awareness Scale

French version

<u>Consigne</u> Parmi les questions suivantes, merci d'indiquer pour chacune d'elle la réponse qui vous correspond le mieux.

<u>Modalités de réponse</u>

1 = Ne me correspond pas du tout
2 = Me correspond très peu
3 = Me correspond peu
4 = Me correspond moyennement
5 = Me correspond assez
6 = Me correspond beaucoup
7 = Me correspond fortement

**Questions**	**Réponse**						
	**1**	**2**	**3**	**4**	**5**	**6**	**7**
1. J'ai besoin d'être très concentré(e) pour prendre conscience de ma posture corporelle	□	□	□	□	□	□	□
2. Quand je me tiens dans une mauvaise posture corporelle, souvent je ne la remarque pas avant qu'elle devienne douloureuse	□	□	□	□	□	□	□
3. Quand je suis assis(e), j'ai souvent tendance à m'avachir	□	□	□	□	□	□	□
4. Quand je me concentre sur une activité spécifique, je prends souvent une posture corporelle particulière sans m'en rendre compte	□	□	□	□	□	□	□
5. Il m'est difficile de prendre consciemment une posture corporelle particulière	□	□	□	□	□	□	□
6. Lorsque je travaille, je vérifie régulièrement ma posture corporelle	□	□	□	□	□	□	□
7. Au travers de ma posture corporelle, je peux intentionnellement modifier l'impression que je donne aux autres	□	□	□	□	□	□	□
8. Tout au long de la journée, je suis en permanence conscient(e) de la façon dont je suis assis(e) ou debout	□	□	□	□	□	□	□
9. Je suis souvent conscient(e) / me rends souvent compte de ma posture corporelle, que je sois assis(e) ou debout	□	□	□	□	□	□	□
10. Même si je suis focalisé(e) sur quelque chose, je suis en permanence conscient(e) de ma posture corporelle	□	□	□	□	□	□	□
11. Au travers de ma posture corporelle, je peux contrôler consciemment mon humeur	□	□	□	□	□	□	□
12. Je remarque si ma posture corporelle est bonne pour moi, ou non, seulement quand je me concentre dessus	□	□	□	□	□	□	□

<u>Calcul des scores</u>

- Dimension *Besoin de réguler son attention pour prendre conscience de sa posture corporelle* : addition après inversion des scores obtenus pour les items 1, 2, 3, 4, 5, 12
- Dimension *Aisance/familiarité avec la conscience de sa posture corporelle* : addition des scores obtenus pour les items 6, 7, 8, 9, 10, 11

Multidimensional Assessment of Interoceptive Awareness Version 2 (MAIA-2)

French version

Consigne Parmi les questions suivantes, merci d'indiquer pour chacune d'elle la réponse qui vous correspond le mieux.

Questions	Réponse					
	0<br>Jamais	1	2	3	4	5<br>Toujours
1. Lorsque je suis tendu(e), je perçois où la tension se situe dans mon corps	☐	☐	☐	☐	☐	☐
2. Lorsque je me sens mal dans mon corps, je le remarque	☐	☐	☐	☐	☐	☐
3. Je perçois dans quelle partie de mon corps je me sens à l'aise	☐	☐	☐	☐	☐	☐
4. Je perçois les changements de ma respiration, par exemple lorsqu'elle ralentit ou s'accélère	☐	☐	☐	☐	☐	☐
5. Je ne perçois pas ou j'ignore les tensions physiques ou l'inconfort jusqu'à ce qu'ils deviennent importants	☐	☐	☐	☐	☐	☐
6. Je me détache des sensations d'inconfort	☐	☐	☐	☐	☐	☐
7. Quand je ressens une douleur ou un inconfort, je mobilise toutes mes forces pour la(le) surmonter	☐	☐	☐	☐	☐	☐
8. J'essaie de ne pas tenir compte de la douleur	☐	☐	☐	☐	☐	☐
9. J'éloigne de moi les sensations d'inconfort en me concentrant sur autre chose	☐	☐	☐	☐	☐	☐
10. Quand je ressens des sensations corporelles désagréables, je fais autre chose de façon à ne pas avoir à les ressentir	☐	☐	☐	☐	☐	☐
11. Lorsque je ressens une douleur physique, cela m'inquiète	☐	☐	☐	☐	☐	☐
12. Je commence à m'inquiéter dès que je ressens de l'inconfort	☐	☐	☐	☐	☐	☐
13. Je peux percevoir une sensation corporelle déplaisante sans m'en inquiéter	☐	☐	☐	☐	☐	☐
14. Je peux rester calme et ne pas m'inquiéter quand je ressens des sensations d'inconfort ou de la douleur	☐	☐	☐	☐	☐	☐
15. Quand j'ai une sensation d'inconfort ou de douleur, je n'arrive pas à penser à autre chose	☐	☐	☐	☐	☐	☐
16. Je peux prêter attention à ma respiration sans être distrait(e)par ce qu'il se passe autour de moi	☐	☐	☐	☐	☐	☐
17. Je peux rester conscient(e) de mes sensations corporelles internes même lorsqu'il se passe beaucoup de choses autour de moi	☐	☐	☐	☐	☐	☐
18. Lorsque je parle avec quelqu'un, je peux porter attention à ma posture	☐	☐	☐	☐	☐	☐
**Questions (suite)**	**Réponse**					
	0<br>Jamais	1	2	3	4	5<br>Toujours

19. Je peux rediriger mon attention sur mon corps si je suis distrait(e)	☐	☐	☐	☐	☐	☐
20. Je peux rediriger mon attention depuis mes pensées vers mon corps (vers mes sensations corporelles)	☐	☐	☐	☐	☐	☐
21. Je peux maintenir l'attention sur l'ensemble de mon corps même lorsque j'ai une douleur ou un inconfort dans une partie de mon corps	☐	☐	☐	☐	☐	☐
22. Je suis capable de me concentrer sur mon corps dans sa globalité	☐	☐	☐	☐	☐	☐
23. Lorsque je suis en colère, je perçois des changements dans mon corps	☐	☐	☐	☐	☐	☐
24. Quand quelque chose ne va pas dans ma vie, je peux le ressentir dans mon corps	☐	☐	☐	☐	☐	☐
25. Après un moment apaisant, je remarque des changements dans mon corps	☐	☐	☐	☐	☐	☐
26. Quand je me sens bien, je remarque que ma respiration devient facile et fluide	☐	☐	☐	☐	☐	☐
27. Quand je me sens heureux(se)ou joyeux(se), je perçois des changements dans mon corps	☐	☐	☐	☐	☐	☐
28. Lorsque je me sens bouleversé(e), je peux retrouver le calme en moi	☐	☐	☐	☐	☐	☐
29. Lorsque je focalise mon attention sur mon corps, je ressens de l'apaisement	☐	☐	☐	☐	☐	☐
30. Je peux utiliser ma respiration pour réduire mon stress	☐	☐	☐	☐	☐	☐
31. Lorsque je suis pris(e) dans mes pensées, j'arrive à m'apaiser en me concentrant sur mon corps ou sur ma respiration	☐	☐	☐	☐	☐	☐
32. Mes sensations corporelles me renseignent sur mon état émotionnel	☐	☐	☐	☐	☐	☐
33. Lorsque je suis préoccupé(e), je prends le temps d'explorer mes sensations corporelles	☐	☐	☐	☐	☐	☐
34. Mes sensations corporelles m'aident à savoir ce que je dois faire	☐	☐	☐	☐	☐	☐
35. Je me sens à l'aise dans mon corps	☐	☐	☐	☐	☐	☐
36. Je me sens en sécurité dans mon corps	☐	☐	☐	☐	☐	☐
37. J'ai confiance en mes sensations corporelles	☐	☐	☐	☐	☐	☐

Calcul des scores : voir https://osher.ucsf.edu/research/maia
